# Intelligent scaling for 6G IoE services for resource provisioning

**DOI:** 10.7717/peerj-cs.755

**Published:** 2021-10-26

**Authors:** Abdullah Alharbi, Hashem Alyami, Poongodi M, Hafiz Tayyab Rauf, Seifedine Kadry

**Affiliations:** 1 Department of Information Technology, College of Computers and Information Technology, Taif University, Taif, Saudi Arabia; 2 Department of Computer Science, College of Computers and Information Technology, Taif University, Taif, Saudi Arabia; 3 College of Science and Engineering, Hamad Bin Khalifa University, Doha, Qatar; 4 Department of Computer Science, Faculty of Engineering & Informatics, University of Bradford, Bradford, United Kingdom; 5 Faculty of Applied Computing and Technology, Noroff University College, Kristiansand, Norway

**Keywords:** Intelligent scaling, 6G enabled Internet of Everything, Resource enhancement, 5G, Data science, Machine learning, Deep learning

## Abstract

The proposed research motivates the 6G cellular networking for the Internet of Everything’s (IoE) usage empowerment that is currently not compatible with 5G. For 6G, more innovative technological resources are required to be handled by Mobile Edge Computing (MEC). Although the demand for change in service from different sectors, the increase in IoE, the limitation of available computing resources of MEC, and intelligent resource solutions are getting much more significant. This research used IScaler, an effective model for intelligent service placement solutions and resource scaling. IScaler is considered to be made for MEC in Deep Reinforcement Learning (DRL). The paper has considered several requirements for making service placement decisions. The research also highlights several challenges geared by architectonics that submerge an Intelligent Scaling and Placement module.

## Introduction

The technological advancement of modern civilization has promoted 5G networks in various fields such as drones, intelligent devices, augmented and virtual reality, smart home appliances, and many interconnected IoT devices in the industrial and medical fields. Though the 5G network shows significant promises, experts are promoting the need to implement a 6G network to improve the Artificial Intelligence and Internet of Everything Devices based learning (Vannithamby & Talwar, 2017). Mobile Edge Computing (MEC) provides users with low communication latency 5G services, and they are the building blocks for Abdallah, Saab & Kassas (2018); Poongodi et al. (2021); Poongodi et al. (2019); 6G architecture, which is gaining popularity due to the increased need for quick services.

The increasing number of heterogeneous services of IoE devices, the changing demand of users, and the limitation of MEC have made it evident that there is a need for resource management for IoT service and MEC servers used for 5G and 6G technologies Al-Sharif et al. (2017). Using manual scaling services such as autonomous driving, network management of vehicles, and automated aerial vehicles for a sustainable future (Saad, Bennis & Chen, 2019). This research paper aims to develop an efficient mechanism for resource scaling along with service placement with the help of automation through AI technology that can be used in various applications Ali et al. (2021). The new solution needs to combine vertical and horizontal scaling for more effective resource management (Yang et al., 2020). The existing auto-scaling solutions lack a model that can help predict the change in service demand.

**Limitations of the current auto-scaling solution are:**

• The current auto-scaling solution does not have a stable solution for predicting change in demand or proper resource management for applications that run on MEC servers Arabnejad et al. (2017).
• The existing model neither has nor explored the availability of resources on MEC servers Benifa & Dejey (2019); Poongodi et al. (2020a); Poongodi et al. (2020b).
• The current scaling solution also does not support multi-application scaling.
• There are not many studies on the solution about scalable resources for significant MEC clusters Bitsakos, Konstantinou & Koziris (2018).
• To mitigate the challenges, the study will explore Deep Reinforcement Learning for controlling the resource and the possibility of having an artificial intelligence-supported 6G cellular environment Farhat, Sami & Mourad (2020). The author suggested using Dyna-Q, which uses the RL algorithm, but this model cannot be used in real life (Mao et al., 2017). The suggested Markov decision process (MDP) design also causes memory issues as it does not show a way for scaling a more significant amount of inputs Fawaz (2018); Fawaz et al. (2017); Gutierrez-Estevez et al. (2018).

**Contributions.** The research also studies the MDP design of IScaler for predicting user demands. The effectiveness of the IScaler predictions enables proactive decisions.

**This research work contributes to of the following:**

• The development of an efficient architecture that used ISP to improve IScaler, a DRL-based solution.
• The custom DQN algorithm can help in building IScaler (Cao et al., 2020).
• The development of MDP mechanism for developing ISaler that manages the MEC requirements.

Through a series of experiments, the paper will highlight the usage of IScaler for performing optimal auto-scaling solutions.

## Literature Review

### Classical solutions

Intelligent or machine-dependent solutions are not used in classical solutions. The search algorithm used is highly complex, and sometimes the solution keeps waiting for the demand to emerge before making a decision. This form suggests that resource scaling requires a heuristics search algorithm in a cloud environment (Sami & Mourad, 2020). However, a heuristic solution is unable to provide the best solutions.

### Machine learning solutions

Recently, the utilization of machine learning has been promoted by experts to solve problems associated with wireless networks and resource management. The RL (Reinforcement Learning)-based solution is a far better option than the classical machine learning solution as it can perform complex and easy approximations and adapt to environmental changes (Gavrilovska, Rakovic & Denkovski, 2018). The application of RL includes resource management of networks, computer-based resource scaling, and the wireless network’s security. DRL (Deep reinforcement learning) solutions are used for modern vehicles, automated aerial vehicles, and modern computers and cellular network technologies Sami et al. (2020); Scarpiniti et al. (2018); Tesauro (1995); Vohra (2016); Xu, Zuo & Huang (2014). DRL also helps in solving problems related to resource management for network slicing Yang et al. (2020); Kaelbling, Littman & Moore (1996); Kherraf et al. (2019); Kim et al. (2020). RL-based estimations are primarily implemented for content caching, and considering the Markov Decision Process, a Model-Based RL mechanism is developed that is responsible for the scaling decision. The application can also contribute to wrong scaling decisions, and if there is any state space, Kumar, Singh & Buyya (2020); Letaief et al. (2019); Li et al. (2019); it would be challenging to assume the possible transition function.

### Industry-based solutions

**Figure 1:**
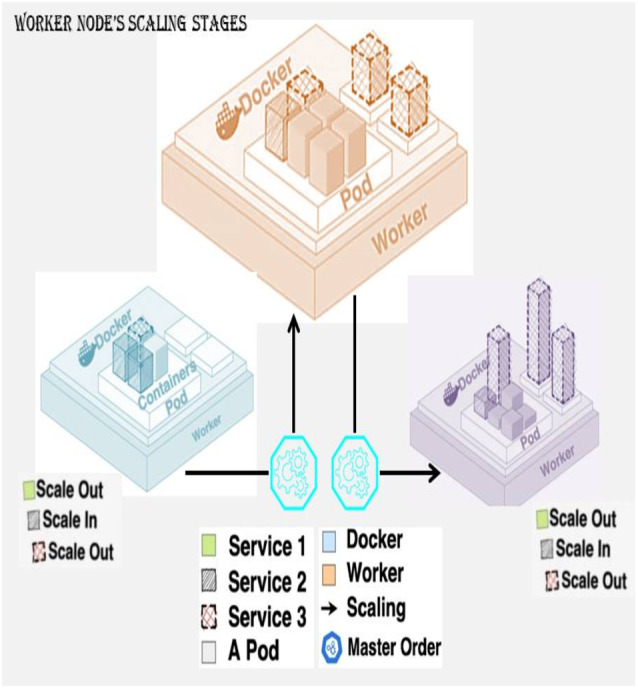
Horizontal and vertical resource scaling problem.

Scaling features are provided mainly by the leading companies, and some of the most popular ones Li et al. (2018); Luong et al. (2019) are Amazon Web Services (AWS), Microsoft Azure, and Google Cloud Platform. The solutions use Kubernetes clustering tools for Procurement of advantage from the scaling and orchestration attributes Malik et al. (2021); Mao et al. (2016); Moati et al. (2014); Poongodi et al. (2021). The prime challenge of these components is that the demand of services like response time or resource load encountered by the user cannot be predictable Saab & Shen (2019); Sadeghi, Sheikholeslami & Giannakis (2017). The solutions heavily rely on manual configurations like that of Azure AutoScale that runs application instances. AWS Auto Scaling is independent of Kubernetes, and the time series prediction that happens can Scales the solicitation instance of precise demands arise (Giordani et al., 2020). Though this method is not reliable because of the inability to capture the demand pattern Rahman et al. (2018); Rauf, Bangyal & Lali (2021), they are heavily used by industries at the service occurrence and levels of the cluster. The architecture of horizontal and vertical resource scaling problems is given in Fig. 1.

## Resource Provisioning and Mec Clusters Architecture

This section provides the architecture for implementing the IScaler technology in MEC clusters that serves as the base for a 6G environment. The container-based cluster architecture uses orchestration technology for resource management (Sami et al., 2021).

### Architecture overview

**Figure 2:**
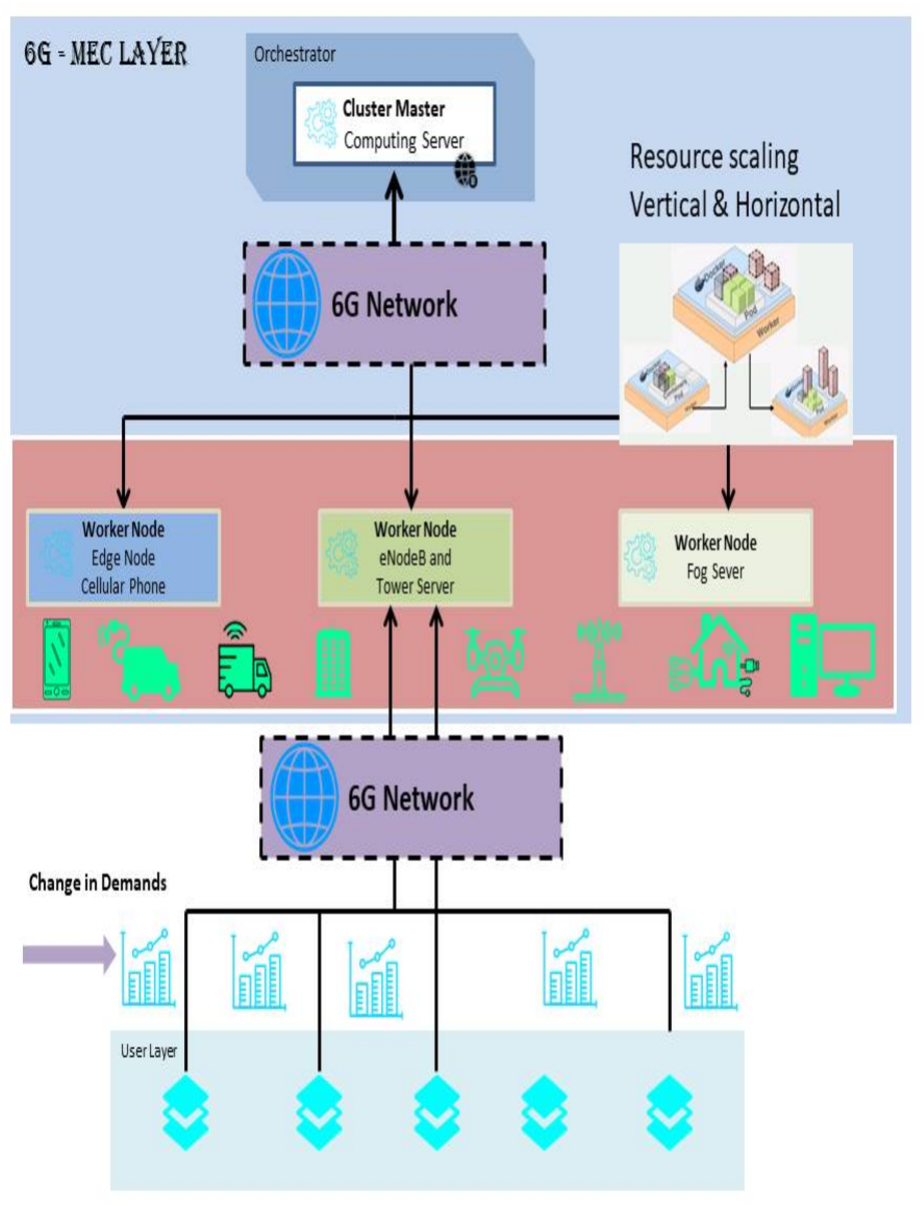
Resource provisioning architecture.

In this research paper, the author has proposed scaling, which is dependent upon the Kubernetes clusters. This technology manages the docker container. Kubernetes could be used for scaling and managing the resources. It also helps in the smooth working of load balancing tasks. Figure 2 shows the common cases covered by this architecture for running a MEC cluster. In the MEC layer, Sami et. al, 2021 IScaler performs scaling, and the scaling decision is executed and hosted by the MEC. The cluster manager node is responsible for running essential Kubernetes elements for managing the cluster and the connection. The master adds and removes worker nodes in the cluster. Furthermore, the master controller installs, removes, and performs physical scaling in the architecture (Alameddine et al., 2019). The failure to reach the fixed result causes rebooting of the function. The worker nodes in the architecture can work on any computing device, from mobile phones to efficient server-based computer engines. The worker nodes support the user’s command and the various changes in the arriving commands. The use of AI can increase the efficacy of the IScaler for load balance.

### Architecture components

Any mistake in the resource scaling can directly affect the host applications, create downtime, and hinder the effectiveness of the QoS and QoE. This section of the paper proposes using an efficient architecture developed by using IScaler to mitigate the errors during the process.

**Caas Module** Various Kubernetes components in the master node are shown by the Container working as a Service (CaaS) module. The cluster orchestration component starts the workers’ management and configuration process. It also updates the logs and highlights the worker nodes’ condition.

**AI based Placement and Scaling** The Intelligent Scaling and Placement or ISP is made up of Optimizer, IScaler, and the Solution Switch. The resource scaling solution provided by IScaler is based on DRL. The issue with the DRL model is that it requires time (Afolabi et al., 2018). To mitigate this problem, the researchers have used a heuristic solution to replace the IScaler. The Optimizer component works as the bootstrapping tool for the IScaler. The researchers have used a threshold-based approach to simplify the process of the ISP.

**Figure 3:**
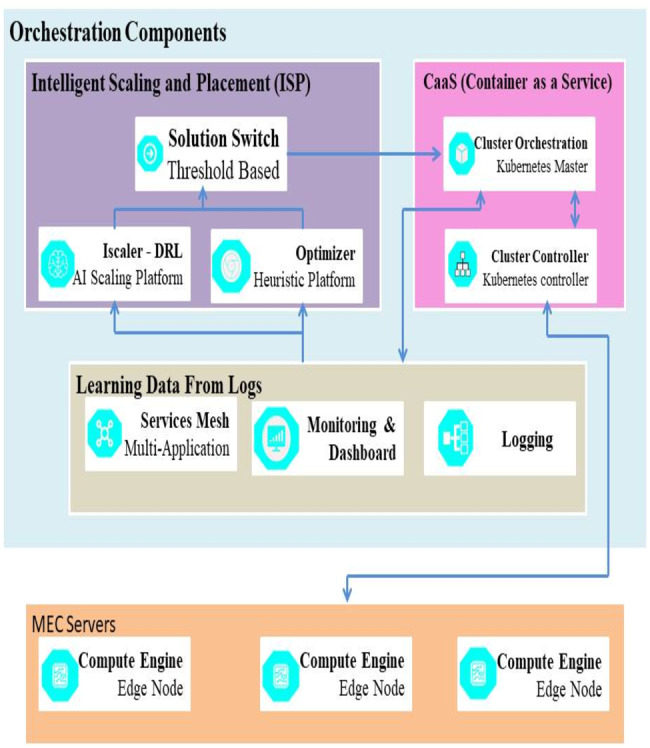
IPS integration in MEC cluster.

**Logs used for Learning Data** The learning data included in the Solution Switch module is used by the Optimizer and IScaler to make decisions. Sever the module manages loads for every server, (Sami et al., 2021) and the hosted micro-services demands are observed. Combining these components’ efficiency helps in learning from the Solution Switch data to improve the IScaler. The framework that includes IPS integration in the MEC cluster is illustrated in Fig. 3.

## Iscaler MDP Formulation

This section provides the detailed MDP formulation for resource scaling to monitor the changing demand of the users and present resources. The IScaler can perform quick learning even when dealing with significant input while using less memory.

### Background

The MDP formulation is a framework used to solve problems using RL. The MDP has tuples $(X,AS,T,\mathcal{B},D)$ in its design which affect can the RL solution scalability and quickness. *X* = {*x*_1_, *x*_2_, ...} is the state space, the action space is *S* = {*s*_1_, *s*_2_, ..., *s*_*l*_}, probability transition matrix is denoted by *T*, cost function is denoted by *F* and discount factor is denoted by *D*.

### State and action spaces

The researcher has denoted the applications of size a that represents the services by *A* = {*A*_1_, *A*_2_, ..., *A*_*a*_}. The set of services of size *a* has been denoted by $Y=\{ {Y}_{1},{Y}_{2},...,{Y}_{k}\} .{Y}_{n}=[{Y}_{n}^{cpu},{Y}_{n}^{mem},{Y}_{n}^{pri},h]$ represents a service *Y*_*n*_ ∈ *Y*. Here, 1 ≤ *n* ≤ *k* and ${Y}_{n}^{mem}$ and ${Y}_{n}^{cpu}$ are respectively the memory requirements and the CPU. If ${Y}_{n}^{pri}$ is high, *Y*_*i*_ is highly recommended for placement and scaling prior to the rest of the services because of its lesser priority. Here, *h* is the application index which means that *Y*_*i*_ ∈ *A*_*h*_. The available hosts of size *v* is represented by *Z* = {*Z*_1_, *Z*_2_, ..., *Z*_*v*_} which are running the service in *Y*. Every host *Z*_*i*_ is defined by ${Z}_{i}=[{Z}_{i}^{cpu},{Z}_{i}^{mem},{Z}_{i}^{dis}]$, where 1 ≤ *i* ≤ *v* and ${Z}_{i}^{dis},{Z}_{i}^{mem},$ and ${Z}_{i}^{cpu}$ are the distance and memory available and the CPU of this host respectively according to the user requests. In every state, the change in demand is denoted by *q*(*u*) for each and every service and *r*(*u*) is a *v* ×2 matrix. We have denoted the normalized available resources of all hosts at *u* and *q*(*u*) is a *k* ×2 matrix. Here, *r*(*u*)_*n*_ represents the line *i* of *r*(*u*) that denotes the average resources for host *Z*_*i*_ , *i.e.,* $r(u)_{i}^{cpu}$ for CPU $r(u)_{i}^{mem}$ for memory Sami et. al, 2021. Here, *p*(*u*) is a *v* ×*k* matrix that is helpful in observing the scaling decision and for the storage of decisions of every host service. Each and every element of *p*(*u*)_*n*,*i*_ has memory allocation and the CPU. Hence, state *x* at *u* in the state space is represented as as:

(1) \begin{eqnarray*}x(u,n,i)=(q(u),r(u),p(u),n,i).\end{eqnarray*}

### Federations conversion and exemplary dynamics

The action space of a specific time step is categorized into several steps. If we denote the current state as *s*, then the state is represented by (*q*(*u*), *r*(*u*), *p*(*u*), *n*^+^, *i*^+^). When *i* = *v*, *i* + becomes 0 and *j* is incremented by 1, it is represented by *n*^+^. Therefore, *i*^+^ = *i* < *v*:*i* + 1?0. Moreover, *n*^+^ = *i* = *v*:*n* + 1?*n*, that means *n*^+^ increases *n* by 1 for the case *i* = *v* and there would be no change in *n*. In case of, internal interaction within a time step, *q* and *r* are constant till the agent does not move to the next time step.

### Cost function

We could calculate the cost function with the help of action taken and the current state of the next state’s agent Sami et. al, 2021. The chief function of IScaler is to obtain the best course of action regarding the current state, which would surely result in minimum cost. The objects of the research are

• Minimizing application cost
• Minimize available resources overload
• Containers priority cost could be minimized
• Cost of other objects could be minimized

Let us assume that a cost is represented as *B*(*x*(*u*-1), *s*(*u*)|*x*(*u*)) helps to evaluate the scaling decision.

• **Minimizing the Application Load**
  The cost of fulfilling the resource requirements of the given application for both memory and CPU are required to be considered. CPU cost is denoted as *B*1 in Eq. (2)
  (2) \begin{eqnarray*}\begin{array}{@{}c@{}} \displaystyle {B}_{1}^{cpu}(u)= \frac{\sum _{n=1}^{k} \left( q(u)_{n}^{cpu}-\sum _{i=1}^{v}p(u)_{i,n}^{cpu}\times {Y}_{n}^{cpu} \right) }{\sum _{n=1}^{k}q(u)_{n}^{cpu}} \end{array}\end{eqnarray*}
  $\text{such that}\forall n,{\mathop{\sum }\nolimits }_{i=1}^{v}p(u)_{i,n}^{cpu}\times {Y}_{n}^{cpu}\lt q(u)_{n}^{cpu}$.
  Here, $q(u)_{n}^{cpu}$ is defined as CPU usage for service *n* and ${Y}_{n}^{cpu}$ is termed as the CPU requirement for service *n*. The price of this provision would be zero once the resource requirement is fulfilled.
• **Minimizing the Overload of the Available Resources**
  For this objective function, the proxy is penalized for overusing the Obtainable assets for scaling the decision. Here, *B*_2_ is the cost of this objective. Eq. (3) represents the CPU cost mathematically.
  (3) \begin{eqnarray*}{B}_{2}^{\text{cpu}}(u)= \frac{\sum _{i=1}^{v}\sum _{n=1}^{k} \left( p(u)_{i,n}^{cpu}\times {Y}_{m}^{cpu} \right) -q(u)_{n}^{cpu}}{\sum _{i=1}^{v}{r}_{i}^{cpu}} \end{eqnarray*}
  $\text{such that}\forall i,{\mathop{\sum }\nolimits }_{n=1}^{k} \left( p(u)_{i,n}^{cpu}\times {Y}_{n}^{cpu} \right) \gt q(u)_{n}^{cpu}$.
• **Priority Cost**
  Each service description is assigned a priority level and the Assessment highlights the scaling of provision over others. *C*_3_ denotes the cost of this objective. Equation (4) shows the CPU cost mathematically.
  (4) \begin{eqnarray*}{B}_{3}^{cpu}(u)= \frac{\sum _{n=1}^{k}\sum _{i=1}^{v} \left( q(u)_{n}^{cpu}-p(u)_{i,n}^{cpu}\times {Y}_{n}^{cpu} \right) \times {Y}_{n}^{pri}}{\sum _{n=1}^{k}q(u)_{n}^{cpu}\times {Y}_{n}^{cpu}} \end{eqnarray*}
  $\text{such that}\forall n,{\mathop{\sum }\nolimits }_{i=1}^{v}p(u)_{i,n}^{cpu}\times {Y}_{n}^{cpu}\lt q(u)_{n}^{cpu}$.
• **Minimize distance cost**
  The infrastructure administration is able to add custom objectives to the IScaler cost function. Equation (5) represents *C*_4_ for Diminishing the whole expanse cost.
  (5) \begin{eqnarray*}{B}_{4}(u)= \frac{\sum _{i=1}^{v}m(u)_{i}\times {Z}_{i}^{dis}}{\sum _{i=1}^{v}{Z}_{i}^{dis}} \end{eqnarray*}
  where ${Z}_{i}^{dis}$ is the distance cost of host *Z*_*i*_, and *m*(*u*) is a vector of size *v* and is calculated as follows: ∀*i*, *m*(*u*)*i* = 1 if ∑*n* = 1^*k*^*p*(*u*)*n*, *i* > 0 and 0 otherwise. A normalization factor of $\sum {i=1}^{k}{Z}_{i}^{dis}$ is added. Therefore,
  Our cost function becomes:
  (6) \begin{eqnarray*}\begin{array}{@{}r@{}} \displaystyle \mathcal{B}((x(u-1),a(u))\mid x(u))={\lambda }_{1}\times {B}_{1}(u)+{\lambda }_{2}\times {B}_{2}(u)+\\ \displaystyle {\lambda }_{3}\times {B}_{3}(u)+{\lambda }_{4}\times {B}_{4}(u). \end{array}\end{eqnarray*}
  *λ* ∈ [0, 1] is a weight relates to each cost function. These weights are adjusted depending on the requirements of the application. The weights also consider the nature of the cluster to give specific cost functions more importance over the others. The purpose of this is to minimize $\mathcal{B}((x(u&minus; 1),a(u))&mid; x(u))$.

## AI-based Scaling & Placement (ISP)

### IScaler with use of Deep Re-inforcement Learning

The IScaler tries to interact with different environments related to evaluating the different placement actions that are seen for each container. The specified agent tries to execute the specific actions in a much-encountered manner and effectively builds a proper and effective strategy that will help to adopt the different stochastic demands, especially of the specified users for the services as per the available resources. The transition probability distributions and help to maintain an optimal policy Φ^∗^ that says about the input as well as output of the different actions as per the future cost. The future causes something that is discounted with the help of *γ*, which is mainly controlled by the different current and past states. Let us take 𝔹(*x*(*u* − 1), Φ|*x*(*u*)) Where it says about the future discounted cost with the help of important policy like Φ at *u* and the action is *s*(*u*′), Where it is seen that $u&le; {u}^{&prime; }&le; \mathcal{U}$, The final equation happens to be in the episode of 𝔹(*x*(*u* − 1), Φ|*x*(*u*)) follows as:

(7) \begin{eqnarray*}\mathbb{B}(x(u-1),\Phi )=\sum _{{u}^{{^{\prime}}}=u}^{U}{\gamma }^{{u}^{{^{\prime}}}-u}\mathcal{B} \left( x \left( {u}^{{^{\prime}}}-1 \right) ,s \left( {u}^{{^{\prime}}} \right) \mid x \left( {u}^{{^{\prime}}} \right) \right) .\end{eqnarray*}

Here optimal action is denoted as *W*^∗^(*s*, *a*) and it says about the function that is being minimised with the help of selected strategy as per below equation:

(8) \begin{eqnarray*}\begin{array}{@{}c@{}} \displaystyle {W}^{\ast }(x,s)=\min _{\Phi }\mathbb{Y }[\mathbb{B}(x(u-1),\Phi )] \end{array}\end{eqnarray*}

where*x*(*u* − 1) = *x*, *s*(*u*) = *s*.

Furthermore $[x..\mathcal{V }]$ is considered to be the chain of the different states and they are linked by using transitions *T* and interpret about W function following the Eq. (9):

(9) \begin{eqnarray*}{W}^{\ast }(x,s)={\mathbb{Y }}_{{x}^{{^{\prime}}}\in [x]}\hspace*{10.00002pt}\text{[V]} \left[ \mathcal{B}+\gamma \min _{{s}^{{^{\prime}}}}W \left( {x}^{{^{\prime}}},{s}^{{^{\prime}}} \right) \right] .\end{eqnarray*}

Here $\mathcal{B}$ identifies the immediate cost in the eq6 and says about the expected value of the last state. The different forms present for RL specifically for the optimal action are being updated with the help of Bellman equation this is given as follows.

(10) \begin{eqnarray*}W(x,s):W(x,s)+\alpha \left[ \mathcal{B}+\gamma \min _{{s}^{{^{\prime}}}}W \left( {x}^{{^{\prime}}},{s}^{{^{\prime}}} \right) \right] .\end{eqnarray*}

A proper approximation is being provided for the different queue functions in a very close manner where *W*^∗^ can be observed in *W*^∗^(*x*, *s*) ∼ *W*(*x*, *s*, *θ*). Taking all the actions effectively, all the surfaces are put forward as per the availability and demands of the resources and the placement of the service. This is followed by the cost of the equation $\mathcal{B}(x(u),s(u+1){|}x(u+1))$.

### Optimizer

A proper evaluation of the MA is the Memetic algorithm with the help of a genetic algorithm helped in the local search process. This is also used for further research work (Sami & Mourad, 2020; Sami, Mourad & El-Hajj, 2020).

Proper utilization of the resources is related to management and acting process done with the help of optimizers and IScaler. Different formulas are used for the hosts and are used for the different available sets. Solution switch is also used to better understand, as in Eq. (6), and the implementation can be effectively found (Sami & Mourad, 2020).

## Experiments and Evaluation

A proper experimental setup can be observed so that better experimentation can be possible with the help of the proposed IScaler. This provides advantages in the recent time with the help of an optimizer in ISP. Some of the objectives are

• To study the DRL model convergence and understand the different multi-application that is in context and understands the resources.
• To highlight the different advantages of optimizer and the phrase involved in IScaler.
• Comparing the different performances of IScaler and the model based algorithm of RL (Rossi et al., 2020).

### Experimental setup

**Figure 4:**
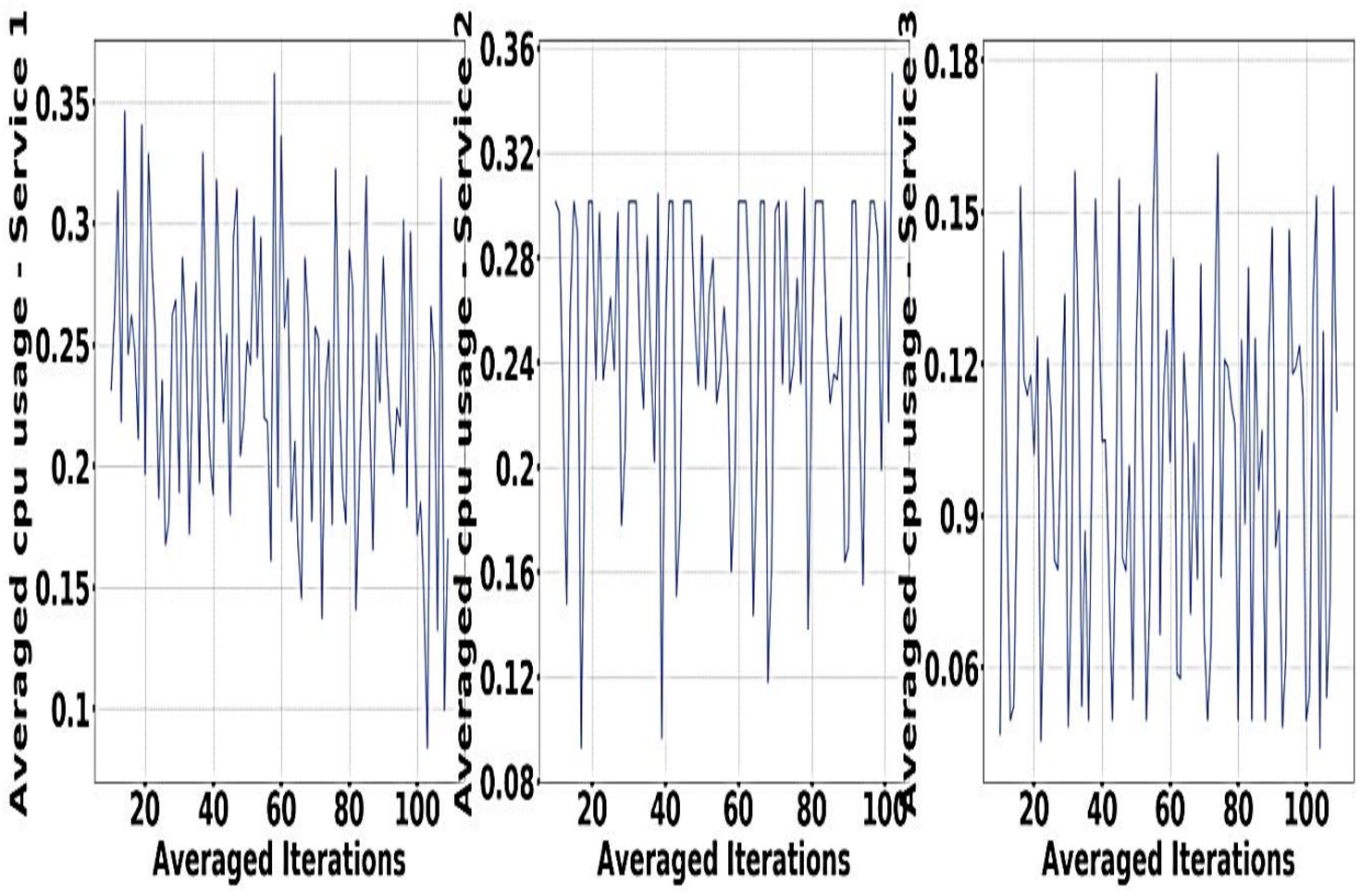
Resource demand of GCT service.

In order to meet different objectives of experiment in an effective manner the algorithm of DRL is being used for the design that is being proposed, that is MDP Building IScaler. 32GB RAM is used with Nvidia Quadro P620 along with GPU training (Sami & Mourad, 2020; Sami, Mourad & El-Hajj, 2020). Sine waves for Resource demand of GCT service are represented in Fig. 4.

**Figure 5:**
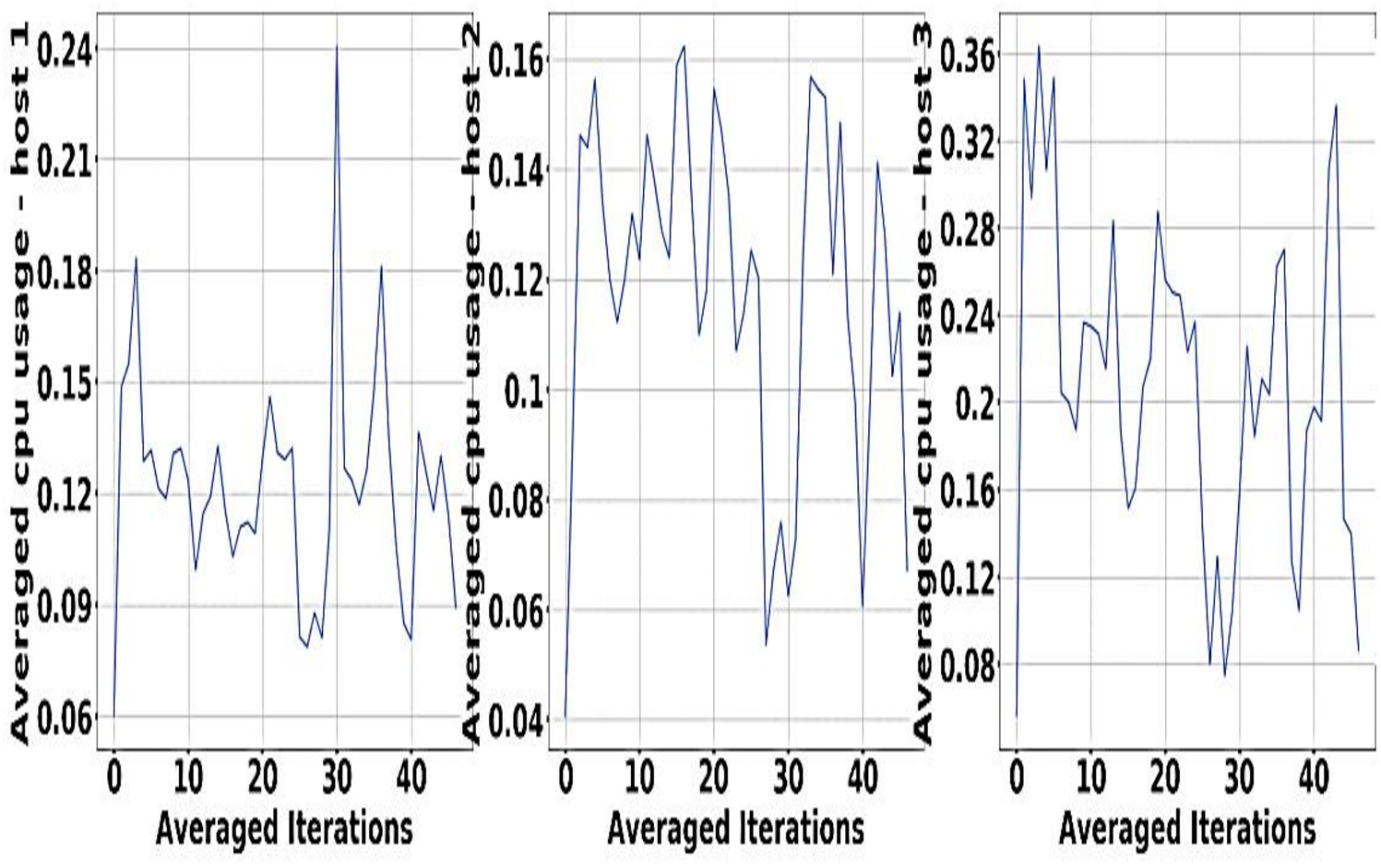
Available resources for host GCT.

**Figure 6:**
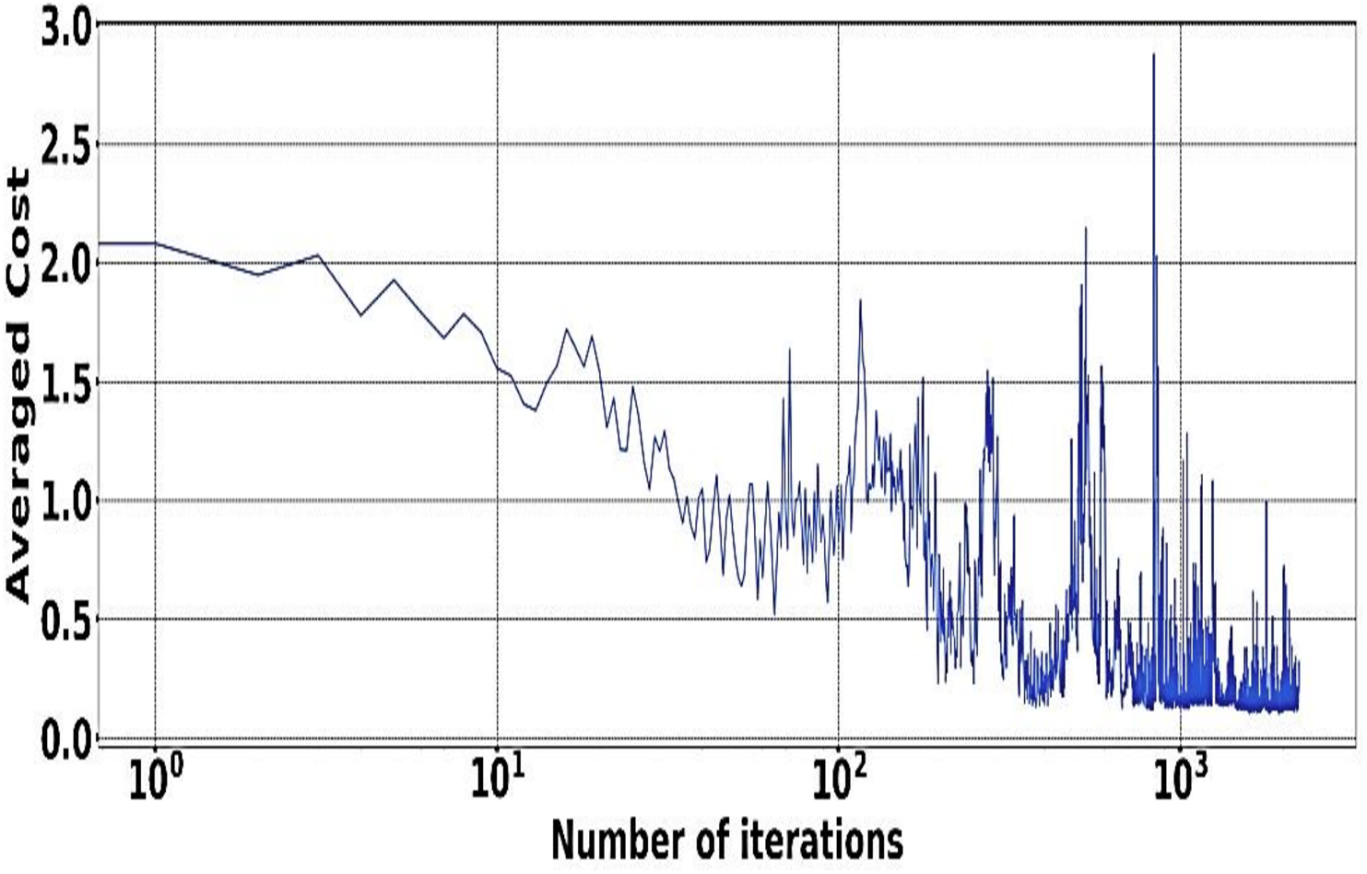
IScaler convergence.

**Figure 7:**
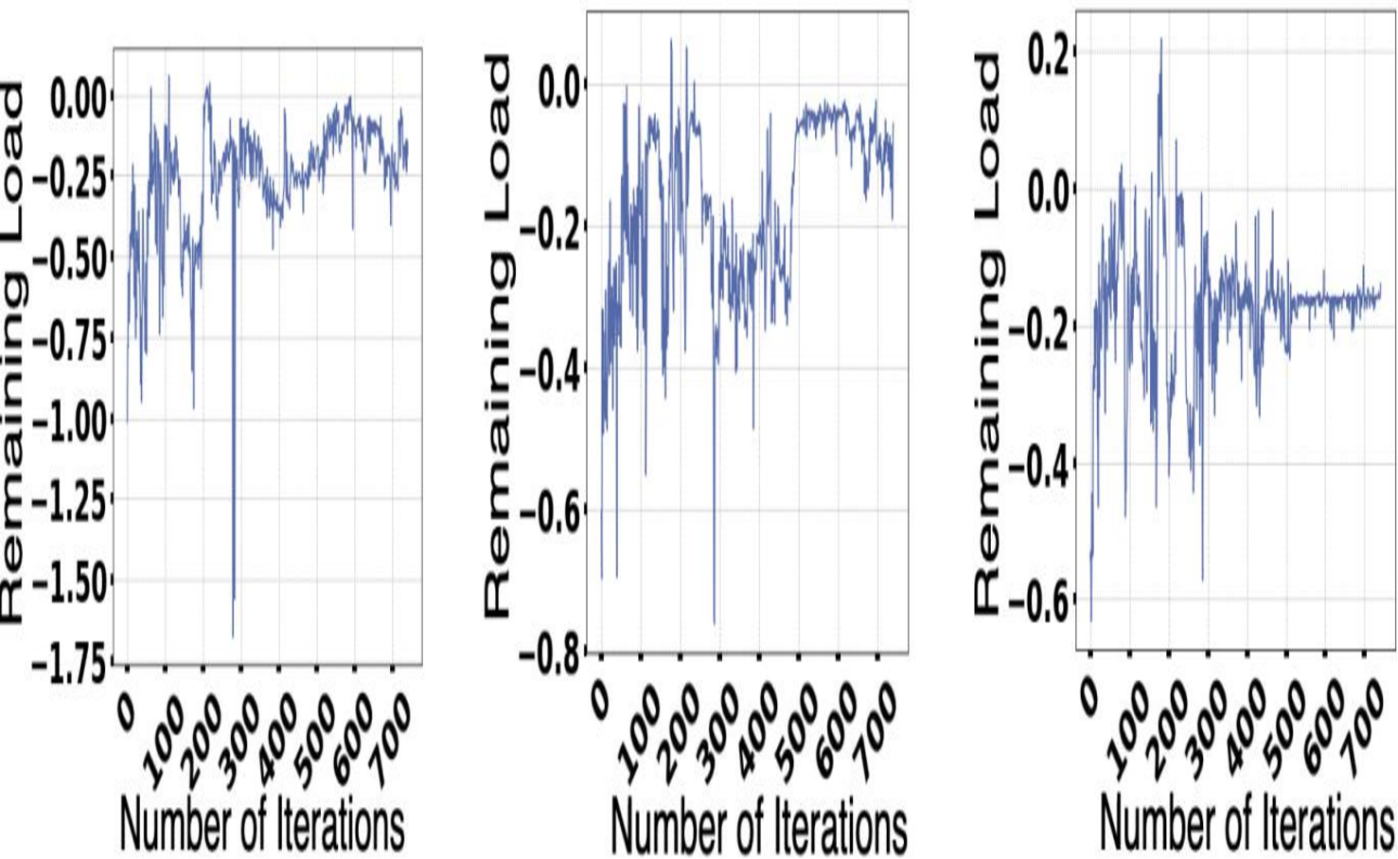
Actual demand and offered resources differences.

The experiment is done with the help of Google Cluster usage (Verma et al., 2015). The different databases and data sets used for the physical machines are being grouped correctly in a particular cell. Solution switch saw to run correctly and help the workers node significantly (Abadi et al., 2016). Figures 5 and 6 provides a better understanding of the different demand resources, and the curve tries to understand the increased demand within the workers. Figure 7 shows actual demand and offered resources differences.

**Figure 8:**
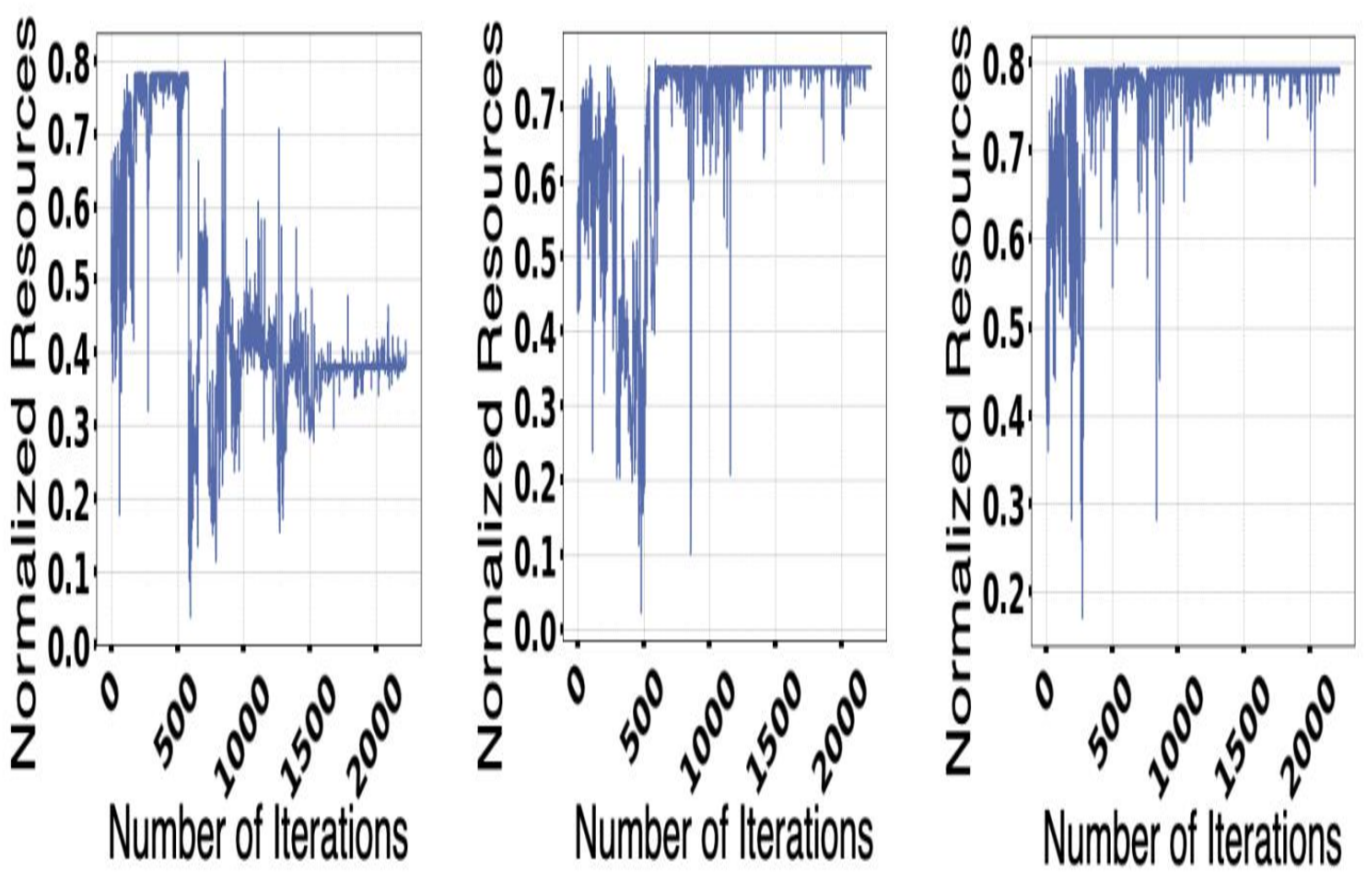
Available resources.

In Fig. 8, proper coverage is provided concerning the different available resources and their change with the host. Elements that are considered high priority are considered, and the amount available for the resources for the different hosts is also taken into account for the experiment.

**Figure 9:**
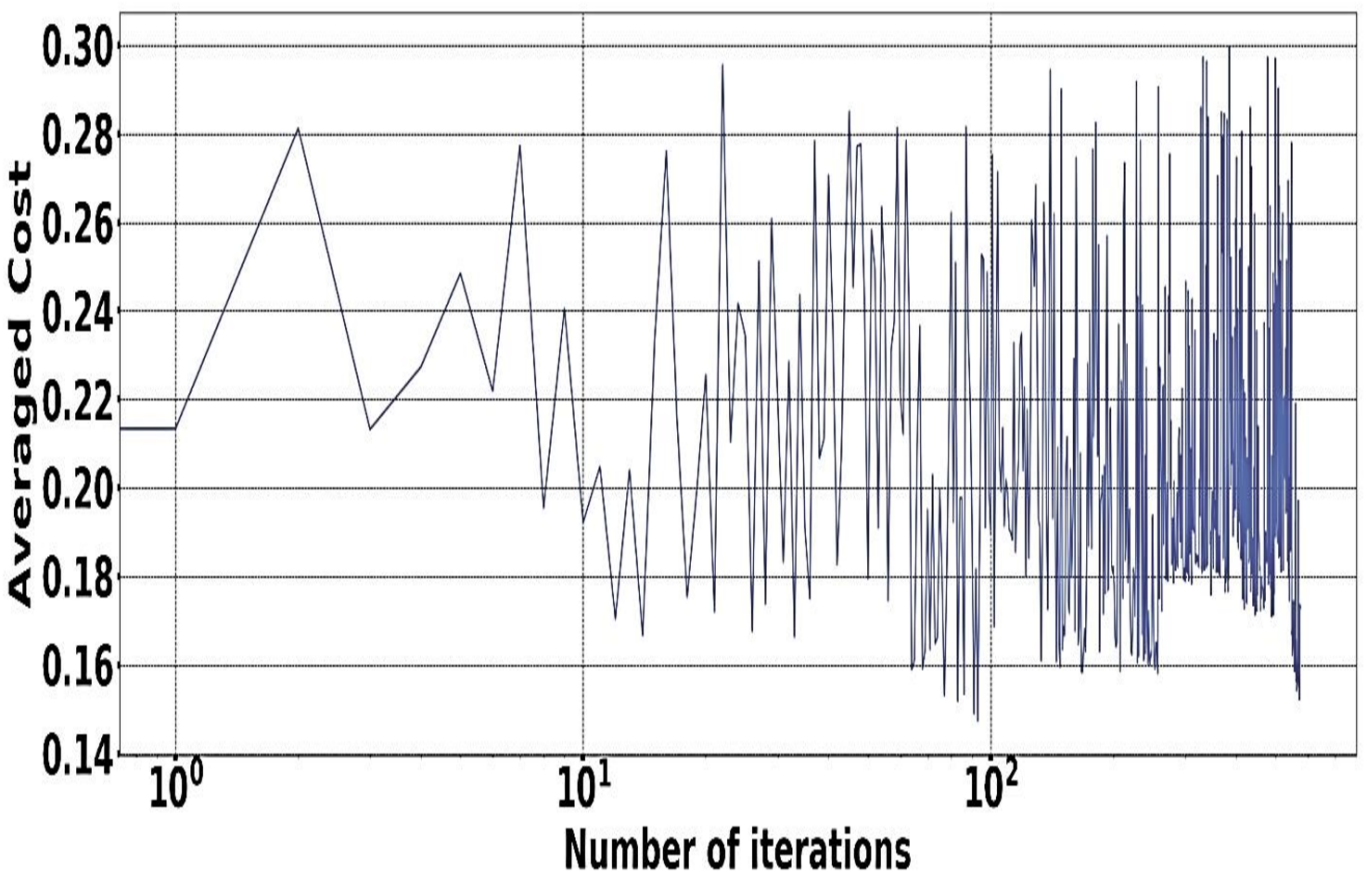
ISP performance.

It always provides a better real-life scenario of the various market demands and is essential for the cluster. All this provides benefits for the experiment and helps in understanding the performance of the scaling services. Figure 9 represents ISP performance on several iterations.

### Model of multi-application convergence

**Figure 10:**
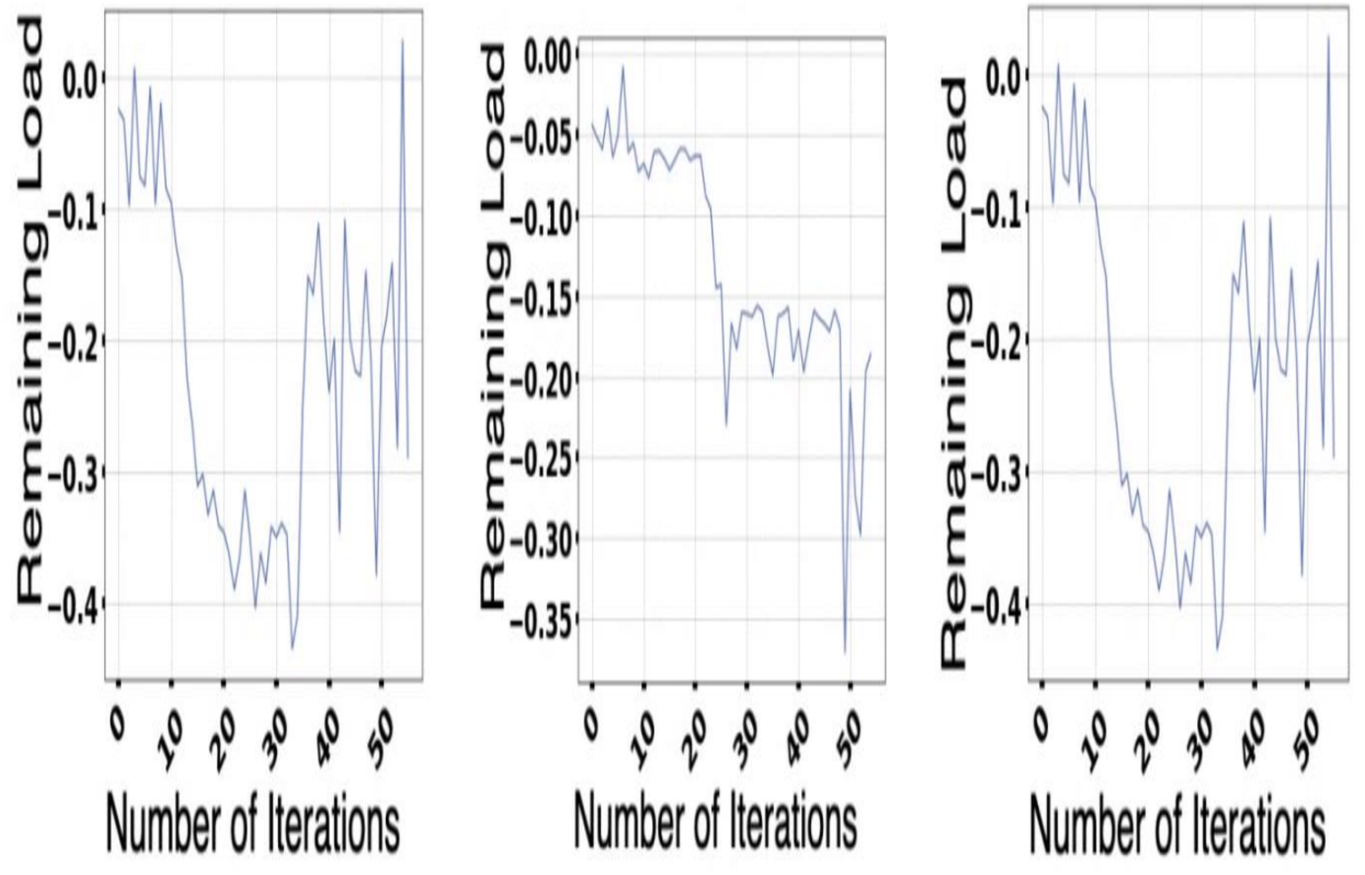
Each services for the resource load.

**Figure 11:**
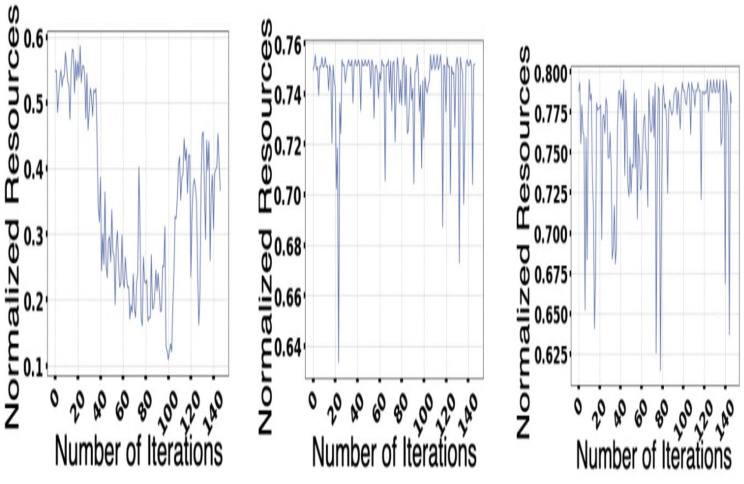
Resource available.

The demand and resources are taken into consideration along with their availability with the help of the converters model as per the different cost values provided as per the decision-making process. Different plot variations can be observed, considering the average cost, and the different graphs provide better log arithmetic skills so that a better visualization can be possible with better agent performance. The different available resources present with time are essential for each host, As given in Fig. 8 and provides a zero value for the different host resources. Each service for the resource load is represented in Fig. 10, where available resources are illustrated in Fig. 11.

### ISP performance

The different figures provide a better understanding of the elements that are related to ISP performances. It says about the different replacements seen for the IScaler and helps in the decision-making process. The result provides an average of the different costs of the resources that are taken as inaccurate. The solution switch also helps to elevate the decision-making that improves the availability of the overall resource. A proper setting is being made that helps understand the behavior of the solution switch and provides an understanding of the average cost of the different variable resources. The different figures provided say about the available resources and the load resources and help to minimize the distance created by the decision of Iscaler. It is also observed that the limitation of an optimizer is that it is often unable to take some of the proactive decisions that are essential for different situations.

### IScaler Vs. Model based scaling

**Figure 12:**
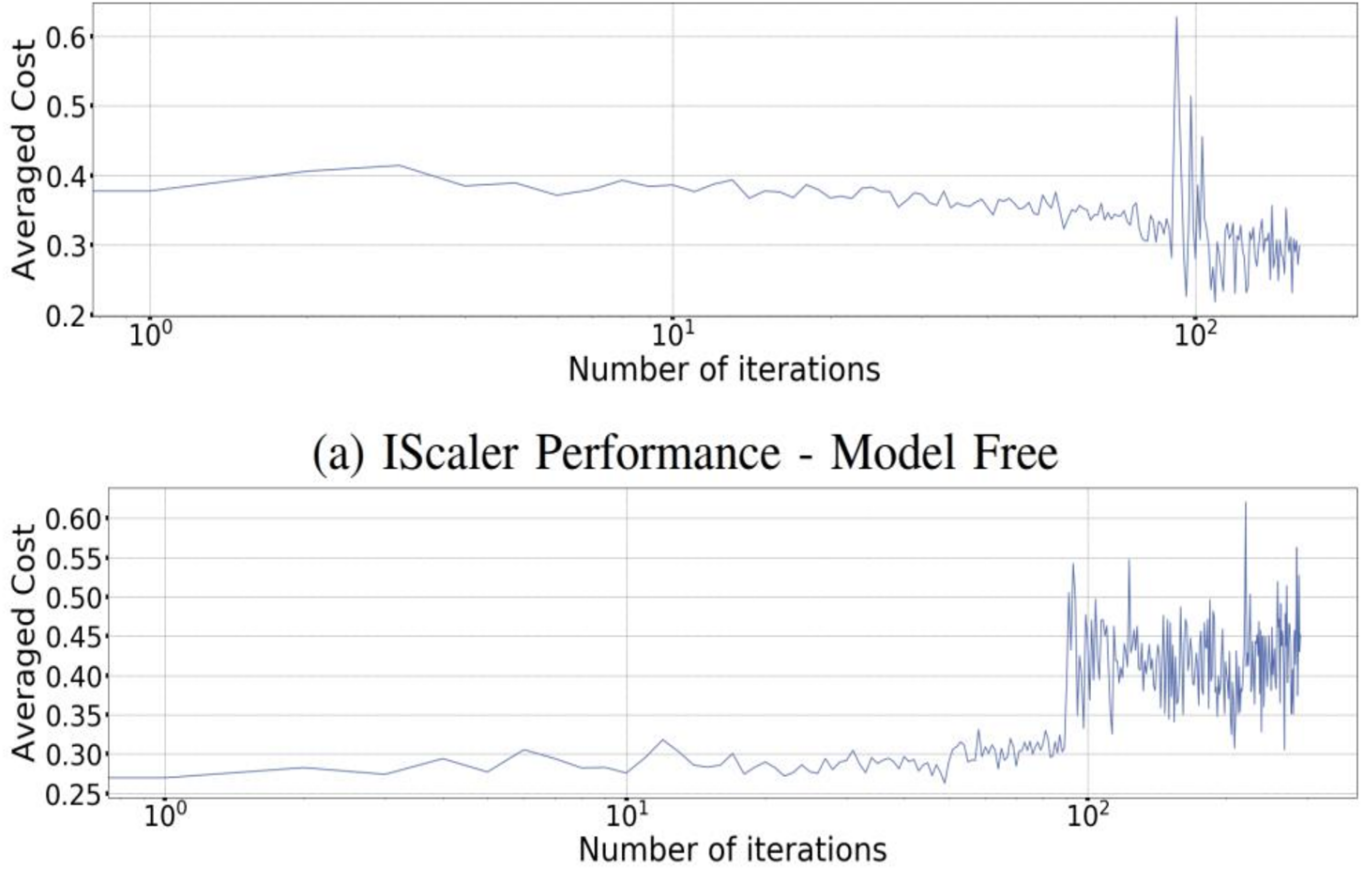
Dyna-Q and IScaler performances.

The different vertical and horizontal resources present for the application of scaling often helps to reinforce the model-based application to a more significant extent. This experiment has tried to replicate the different elements of the Dyna-Q model present for this experiment’s case. This has tried to use the different tabular that are present in the model and have incorporated some critical available resources for the case of Q-learning. The different matrices are used for the case of Dyna-Q, and Fig. 12 shows that a significant change and a dynamic environment can be observed for the case of resources that are primarily available for the cluster. The other update shown in Fig. 12 provides new samples and an understanding of the drastically changing situation with the help of effective reward and the significant signals. It also provides some approximation of the elements that are present for the cluster.

## Conclusion and Future Work

Moving towards a better hosting, development, and proper management of the different services of the new era that are backed up by 5G and 6G, the need for availability of the different computer resources that MEC provides. Because of the limited amount of resources with MEC, the infrastructure for the different applications is considered a challenge, especially for the cellular network. In this paper, keeping an eye, a suggestion about IScaler is being made. IScaler is considered one of the multi-application that help in scaling and can overcome the different challenges necessary for the dynamic environment. It is seen that the DRL-based applications that involve 5G or 6G are costlier. Here some proposals are made for the optimizer, IScaler, and long solution switch. However, it is also evident that ISP is efficient in decision making in (1) performing some intelligence with the help of multi-application decision, (2), during the use of IScaler optimizer is used, (3) understanding the specific ability of IScaler, so that can be used with some existing solutions like model-based scaling.

## Additional Information and Declarations

### Data Availability

The following information was supplied regarding data availability: The data is available at GitHub: https://github.com/pookuttym/GoogleCloudStorage-Traves-2019/find/main
The code is available at GitHub: https://github.com/pookuttym/Intelligent-Scaling_6G_IoE-Script.
